# Modeling the P2X7 receptor: a comparative analysis of conventional methods and AlphaFold

**DOI:** 10.3389/fphar.2026.1685425

**Published:** 2026-04-21

**Authors:** Lauro Miranda Lima, Natiele Carla da Silva Ferreira, Luiz Anastacio Alves

**Affiliations:** Laboratory of Cellular Communication, Oswaldo Cruz Institute, Oswaldo Cruz Foundation, Rio de Janeiro, Brazil

**Keywords:** AlphaFold, docking, homology modeling, P2X7, protein structure

## Abstract

**Background:**

P2X7 is a purinergic receptor involved in inflammation, pain, and neurodegeneration and is an important pharmacological target, with no drugs approved for clinical therapy. Given the limited availability of crystallized human P2X7 (hP2X7) receptor structures, this study evaluates the structural fidelity of models generated through conventional homology modeling and advanced deep learning approaches using AlphaFold, aiming to support structure-based drug discovery.

**Methods:**

The hP2X7 receptor was modeled through conventional homology-based methods using SWISS-MODEL guided by template and sequence alignment. Additional structural models were generated by AlphaFold 2 (AF2) and AlphaFold 3 (AF3). All four resulting models were assessed for confidence levels at orthosteric and allosteric binding sites and employed in docking simulations with adenosine triphosphate (ATP) and JNJ47965567 to evaluate their suitability for capturing key ligand-receptor interactions.

**Results:**

Two rat P2X7 (rP2X7) receptor structures were selected as templates for modeling the hP2X7 protein: one in an ATP-bound open conformation (Protein Data Bank, PDB ID: 6U9W) and the other in an antagonist-bound closed state (PDB ID: 8TRB). These templates yielded two homology-based models, Q99572–6U9W and Q99572–8TRB, with scores of 0.71 and 0.77, respectively, indicating great structural quality. On the other hand, AF2 and AF3 generated hP2X7 receptor models with scores of 0.841 and 0.82, respectively, reflecting high prediction confidence. However, a key limitation of AlphaFold algorithms is their ability to generate P2X7 receptor structures exclusively in the closed state, restricting their ability to model ligand-bound open conformations. Docking simulations suggest that models generated using either AF2 or comparative modeling exhibit enhanced interaction profiles with key residues at both the orthosteric and allosteric binding sites of the P2X7 receptor.

**Conclusion:**

This study compared AF2 and AF3 with conventional modeling techniques for constructing hP2X7 receptor models and revealed that, compared with AF3, AF2 generated high-confidence models with strong ligand-binding potential for virtual screening (VS), despite the latter effectively modeling protein–ligand complexes. Traditional modeling remains crucial for improving accuracy in flexible or poorly resolved regions. Protein modeling advancements hold the potential to revolutionize VS, accelerating the discovery of novel P2X7 receptor antagonists.

## 1 Introduction

The P2X7 receptor is a transmembrane protein broadly distributed across various mammalian cell types, with predominant expression in immune and glial cells (Skaper et al., 2010). Functionally, P2X7 receptor assembles as a homotrimeric receptor composed of three identical subunits. The polypeptide chain of each monomer comprises 595 amino acid residues and is structurally organized into distinct functional domains: (i) an intracellular N-terminal domain containing 26 residues; (ii) a large extracellular domain of 282 residues, which accommodates both orthosteric and allosteric ligand-binding sites; (iii) two transmembrane domains (TM1 and TM2), each formed by α-helical segments of approximately 24 residues; and (iv) an extended cytoplasmic C-terminal domain consisting of 239 residues (Di Virgilio et al., 2017).

This receptor is physiologically activated by extracellular adenosine triphosphate (ATP), which triggers the opening of an ion channel and allows the influx of Ca^2+^ and Na^+^, as well as the efflux of K^+^, according to the electrochemical gradient. High concentrations of ATP (>100 µM) and prolonged exposure to this agonist result in a second stage of P2X7 receptor activation, characterized by the formation of a pore in the cytoplasmic membrane, permitting the passage of molecules up to 900 Da (Skaper et al., 2010; Alves et al., 2014; Karasawa and Kawate, 2016). Furthermore, P2X7 receptor activation leads to proinflammatory responses, including inflammasome assembly, the release of cytokines such as interleukin-1β and tumor necrosis factor-α, the generation of reactive oxygen species, the activation of transcription factors such as MAPK and NF-KB, and ultimately, cell death (Lister et al., 2007; Skaper et al., 2010; Jacob et al., 2013; Bartlett et al., 2014).

Numerous studies have correlated P2X7 receptor activation with chronic inflammatory diseases, pain, neurodegenerative disorders, and certain cancers (Skaper et al., 2010; Keystone et al., 2012; Stock et al., 2012; Jacob et al., 2013; Vergani et al., 2013; Fresta et al., 2020). Furthermore, P2X7 receptor inhibition in animal models has been shown to have protective effects against these conditions (Adinolfi et al., 2012; Martin et al., 2019; Sluyter et al., 2023). As a result, several molecules have advanced to phase I and II clinical trials for treating rheumatoid arthritis, chronic inflammatory pain, and depression (Supplementary Table S1), underscoring the therapeutic potential of this receptor in various pathological contexts (Keystone et al., 2012; Stock et al., 2012; Park and Kim, 2017; Recourt et al., 2023).

Despite efforts to develop a selective antagonist for the P2X7 receptor, no drug has yet been approved for clinical use. One factor that may explain this obstacle is the discrepancy in results between preclinical animal studies and human trials, which could be partly attributed to the substitution of leucine (L95) in rats with phenylalanine (F95) in humans (Caseley et al., 2015). The structure of the P2X7 receptor has thus far been experimentally resolved in rats (*Rattus norvegicus*), giant pandas (*Ailuropoda melanoleuca*), chickens (*Gallus gallus*), and, more recently, humans. In light of these advances, computational approaches, including traditional homology modeling and deep learning methods based on attention mechanisms, such as AF2 and AF3, are emerging as powerful tools for constructing reliable models of the predicted structure of the human P2X7 receptor. These predicted structures can be harnessed in VS pipelines to discover novel and highly selective antagonists.

AlphaFold is a cutting-edge computational method designed to predict the three-dimensional structure of proteins. It gained significant attention during the Critical Assessment of Structure Prediction 13 (CASP13) through its initial version, AlphaFold. The subsequent version, AF2, outperformed its initial version in CASP14. Currently, the program is in its third version, AF3, and continues to push the boundaries of structural biology (Karelina et al., 2023; Abramson et al., 2024).

AF2 is a modeling program that takes the target protein sequence as input. The sequence is used to construct a multiple sequence alignment (MSA) by searching genetic databases for sequences similar to the target. The MSA is utilized to obtain coevolutionary relationships between the sequences and to create a pairwise representation for each residue of the target protein. In parallel, the MSA is also used to search for experimentally resolved structures similar to the target protein in structural databases. All these data are processed by two main neural networks: the Evoformer, which uses attention mechanisms to iteratively analyze the interactions between residue pairs and infer distances and relative positions; and the Structure module, which is responsible for using both the target’s primary sequence and the refined information from the Evoformer to construct the protein’s backbone. Finally, the structure module adds the amino acid side chains and adjusts and refines the final positions of the residues in the three-dimensional structure (Jumper et al., 2021).

On the other hand, AF3 was developed with the ambitious goal of accurately predicting different biomolecules, such as amino acids, RNA, DNA, ions, and ligands. AF3 takes the sequences of the biomolecules to be predicted as input. Similar to AF2, the sequences are used to search genetic databases (i.e., proteins and RNA) and structural databases (i.e., proteins). The data obtained from structural databases are used as templates, providing spatial atomic data for the template module. The search of genetic databases is used to form an MSA of the sequences, obtaining coevolutionary data passed to the small MSA module. Other biomolecules are used as individual sequence inputs or as an interaction matrix. These data are fed into the primary module, known as the pairformer. This attention module is responsible for constructing matrices that represent the interactions between the atoms of the input sequences. The information is then forwarded to the diffusion module, which constructs the model by positioning individual atoms according to the interactions identified by the pairformer. As demonstrated in the tests presented in the AF3 publication, even when the model for the prediction of a broader spectrum of biomolecules is generalized, its performance surpasses that of the previous model in predicting the structures of monomeric and multimeric proteins and protein-antibody interactions (Abramson et al., 2024).

Despite significant advancements in homology modeling in recent years, particularly with the use of attention-based neural networks, there is still limited discussion about the application of these methodologies in the search for novel selective antagonists for the P2X7 receptor. In this study, we aimed to evaluate the structural prediction accuracy of the P2X7 receptor using both conventional homology modeling techniques and advanced attention-based approaches employed by AF2 and AF3. Additionally, we investigate the applicability of these models in docking assays, focusing on the discovery of novel P2X7 antagonists.

## 2 Materials and methods

### 2.1 Structural similarity search for the hP2X7 receptor

The hP2X7 receptor sequence was obtained from the UniProt database (available at https://www.uniprot.org/) under the ID Q99572. After the sequence was acquired, a BLAST-p search was performed in the PDB database.

### 2.2 Sequence analysis

To align the hP2X7 receptor sequence (ID: Q99572) and potential template structures for homology modeling, rP2X7 receptor structures were used in both the open (PDB ID: 6U9W) (McCarthy et al., 2019) and closed states (Protein Data Bank, PDB ID: 8TRB) (Oken et al., 2024). The alignment was conducted using the CLUSTAL Omega server, accessible at https://www.ebi.ac.uk/jdispatcher/msa/clustalo (Sievers et al., 2011).

### 2.3 Comparative modeling

Due to the high coverage and sequence identity of the selected templates relative to the target sequence, modeling assays were conducted in the SWISS-MODEL server (available at https://swissmodel.expasy.org/) (Waterhouse et al., 2018). With the 6U9W and 8TRB protein structures as templates, two homotrimeric models of the P2X7 receptor were constructed: Q99572-8TRB (hP2X7_closed) and Q99572-6U9W (hP2X7_open). Both models underwent a relaxation step using the energy minimization tool in Chimera software (Pettersen et al., 2004) to optimize their geometry.

The quality of these models was subsequently rigorously evaluated using the structural assessment tool of SWISS-MODEL, which employs a set of metrics to ensure the accuracy and reliability of the predicted structures. For stereochemical analysis, MolProbity was used to assess the local quality of the model’s geometry, including the backbone conformation and atomic interactions. Additionally, Quality Model Energy Analysis–Distance Constraint (QMEANDisCO) was applied to estimate the quality metrics. This score, which ranges from 0 to 1, reflects the model’s overall quality by comparing it to reference structures, providing a robust measure of its global and local accuracy (Waterhouse et al., 2018). The integrated evaluation of these metrics is crucial for validating the models in structural biology studies.

### 2.4 Modeling with AF2

ColabFold was used to construct the hP2X7 receptor homotrimeric model. ColabFold (available at https://colab.research.google.com/github/sokrypton/ColabFold/blob/main/AlphaFold2.ipynb#scrollTo=ADDuaolKmjGW) is a free online script for building three-dimensional models of protein monomers and multimers. For model construction, AF2-multimers version 3 was utilized, with three cycles and zero tolerance, following the ColabFold recommendations. At the end of this process, five models of the hP2X7 receptor were obtained, and the Rank_1 model was selected for its superior metrics: predicted local distance difference test (pLDDT), predicted template modeling (pTM), predicted alignment error (PAE), and interface-predicted template modeling (ipTM) metrics (Jumper et al., 2021).

These metrics are essential for assessing the quality of the generated models. The pLDDT measures the confidence score for each amino acid residue. Scores above 90 indicate very high confidence, whereas scores between 70 and 90 are considered reliable. Scores less than 50 suggest low confidence. PAE is a global metric that estimates the expected position error between two residues after aligning the model. It is particularly useful for assessing the relative orientation of different protein domains. The pTM score evaluates the overall accuracy of the model compared with that of a real structure, providing a single value between 0 and 1. Finally, the ipTM score is a variant of pTM specifically designed for multimeric complexes, focusing on the quality of the interface between different protein chains. High ipTM values indicate strong confidence in the subunit arrangement and packing at the interaction surface (Jumper et al., 2021; De Salis et al., 2024).

### 2.5 Modeling with AF3

AF3 modeling was performed using the online server available at https://alphafoldserver.com/. To generate the trimeric structure of the hP2X7, three copies of the receptor sequence were provided as inputs, allowing the prediction of the homotrimeric assembly. To model the ATP-bound state, the ATP ligand was explicitly included during the prediction process using the ligand incorporation functionality of the server. Among the generated models, the structure with the highest pTM and ipTM scores was selected for subsequent structural analyses.

### 2.6 Assessment of the confidence levels of orthosteric and allosteric sites in the structural models generated by AF2 and AF3

To assess the confidence levels of the orthosteric and allosteric binding sites in the generated models, a comprehensive literature review was conducted to identify the key residues involved in the interaction between the P2X7 receptor and its corresponding ligands. The selected residues were K64, K66, T189, N292, R294, and K311 for the orthosteric site and F88, F95, F103, M105, F293, and V312 for the allosteric site. Based on the selection of these residues, the pLDDT values were extracted using a custom Python script (available at https://github.com/LauroML/Extrator_pLDDT_AF2_AF3/blob/main/extrator_de_plddt.ipynb). For models generated by AF2, pLDDT scores were obtained directly at the residue level. In contrast, AF3 assigns pLDDT values per atom; therefore, the average atomic values were calculated to represent the pLDDT for each residue. In addition to the preselected residues, the five adjacent residues for each one were also evaluated, aiming to derive a comprehensive global confidence metric at both the residue and site levels.

### 2.7 Model comparison

The Root Mean Square Deviation (RMSD) was employed to compare the models obtained through modeling with their respective templates as well as to assess structural differences among all models (De Salis et al., 2024).

$$RMSD=\sqrt{\frac{1}{N}\sum_{i=1}^{N}{\left(r_{i}-r_{i}^{ref}\right)}^{2}}$$

RMSD was employed to quantify the structural similarity between the computationally predicted models and the experimental reference structure. In the equation, N denotes the total number of paired atoms considered in the comparison, $r_{i}$ represents the Cartesian coordinate vector of atom *i* in the evaluated structure, and $r_{i}^{ref}$ corresponds to the coordinate vector of the same atom in the reference structure.

### 2.8 Redocking assays

Redocking experiments were performed using GNINA version 1.3 to evaluate the ability of the docking algorithm to reproduce experimentally determined ligand binding poses. The rP2X7 crystal structures PDB ID: 8TRB (closed conformation, cocrystallized with the allosteric antagonist JNJ47965567) and PDB ID: 6U9W (open conformation, cocrystallized with ATP) were used as reference systems for the allosteric and orthosteric sites, respectively.

Docking grids were defined using the geometric center coordinates of the cocrystallized ligands (JNJ47965567 and ATP) in their respective reference structures. A grid box of 40 × 30 × 40 Å was employed to fully encompass the binding pocket and surrounding interacting residues. Docking performance was evaluated by calculating the RMSD between the predicted and crystallographic ligand poses, as described in Equation 1.

To address the issue of atomic symmetry, particularly in molecules containing chemically equivalent atoms, a symmetry-corrected RMSD calculation was implemented using the Hungarian algorithm (Kuhn, 1955), also known as the Munkres assignment method. This algorithm was applied to each cost matrix to determine the optimal one-to-one correspondence between atoms of the same chemical element, minimizing the total sum of squared interatomic distances. This procedure prevents artificial inflation of RMSD values resulting from index permutations of symmetry-equivalent atoms.

Redocking accuracy was interpreted according to widely accepted criteria in the literature, whereby RMSD values below 2.0 Å indicate satisfactory reproduction of the crystallographic binding pose.

### 2.9 Docking assays

For the docking assays, the models generated by SWISS-MODEL and AF2 and AF3 were protonated using the PDB2PQR server version 3.6.1 (available at https://server.poissonboltzmann.org/pdb2pqr), adjusting protonation at pH 7.4 with PROPKA. The resulting PQR files were converted back to PDB using PyMOL. The reference ligands were also protonated at pH 7.4 using the Open Babel library.

Docking simulations were conducted using GNINA version 1.3, which employs a rescoring function with the default convolutional neural network (CNN) scoring model. To ensure reproducibility, a fixed random seed (seed −1985) was applied in all the docking runs.

To define the orthosteric binding site, the homology models generated by SWISS-MODEL and the predicted structures obtained from AF2 and AF3 were structurally aligned to the experimental structure of rP2X7 in the open conformation (PDB ID: 6U9W) using PyMOL. The docking grid box was centered on the cocrystallized ATP molecule present in 6U9W structure. A cubic grid box with dimensions of 40 Å × 30 Å × 40 Å was defined to encompass the entire orthosteric binding pocket and surrounding residues relevant for ligand accommodation.

For the allosteric binding site, the same structural models were aligned to the rP2X7 closed-state structure (PDB ID: 8TRB). The docking grid was centered on the cocrystallized allosteric antagonist JNJ47965567 present in the 8TRB structure. Similarly, a cubic grid box with dimensions of 40 Å × 30 Å × 40 Å was employed to fully cover the allosteric cavity and adjacent interaction regions.

All the docking poses were ranked according to GNINA CNN scoring, and the top-ranked conformations were selected for subsequent structural and interaction analyses.

Protein‒ligand interaction analyses were conducted using the Protein‒Ligand Interaction Profiler (PLIP; available at https://plip-tool.biotec.tu-dresden.de/plip-web/plip/index), and the results were subjected to manual visual inspection.

## 3 Results

### 3.1 Construction of hP2X7 models using conventional modeling

To evaluate the performance of models generated by attention-based neural networks, specifically AF2 and AF3, in comparison with models obtained through conventional comparative modeling techniques, we conducted structural modeling of the hP2X7 receptor using different methodologies. For the classical approach, the hP2X7 sequence was retrieved from the UniProt database (ID: Q99572). A BLASTp algorithm was used to search for homologous structures in the PDB. The initial BLASTp search revealed 24 structural entries related to the P2X7 receptor, with the top 10 ranked results summarized in Supplementary Table S2.

The top-rated structures were two apo rP2X7 receptor in the closed state, 6U9V and 8TRB, with resolutions of 2.90 and 2.36 Å, respectively. These structures exhibited 100% coverage of the hP2X7 receptor and approximately 80% identity (Supplementary Table S2). The 5U1L structure, which corresponds to the panda P2X7 receptor (pdP2X7), has the highest identity with hP2X7. However, its coverage is low (57%) since it does not present the C-terminal region of the P2X7 receptor.

Template selection was guided by three primary criteria: (i) highest sequence identity and query coverage with the hP2X7 receptor; (ii) availability of cocrystallized structures with either the physiological agonist (ATP) or an allosteric antagonist captured in the receptor’s native conformation (i.e., open or closed P2X7 channel, respectively); and (iii) high-resolution crystallographic data. Based on these criteria, we selected the 6U9W and 8TRB proteins for modeling assays. These two rP2X7 structures were resolved by cryo-electron microscopy (cryo-EM), with resolutions of 3.30 and 2.36 Å, respectively. The 6U9W structure represents the receptor in an open conformation bound to ATP, whereas 8TRB depicts the closed conformation complexed with the antagonist JNJ47965567. Both templates exhibited 100% sequence coverage relative to the hP2X7 receptor and shared more than 80% sequence identity (Supplementary Table S2).

Sequence alignment was subsequently performed between the hP2X7 receptor (UniProt: Q99572) and the structural models 6U9W (rP2X7_open) and 8TRB (rP2X7_closed). The alignments revealed 478 conserved residues, 82 conserved substitutions, and 40 nonconserved substitutions (Supplementary Figure S1). Notably, compared with the Q99572 sequence, the 6U9W structure has a C-terminal tail containing 14 additional residues, resulting in 14 alignment gaps (Supplementary Figure S1).

Based on the alignment data, comparative modeling of the hP2X7 receptor was performed. High-resolution structures 6U9W (rP2X7_open) and 8TRB (rP2X7_closed) were selected as templates due to their high sequence identity and full coverage of the target sequence.

**FIGURE 1:**
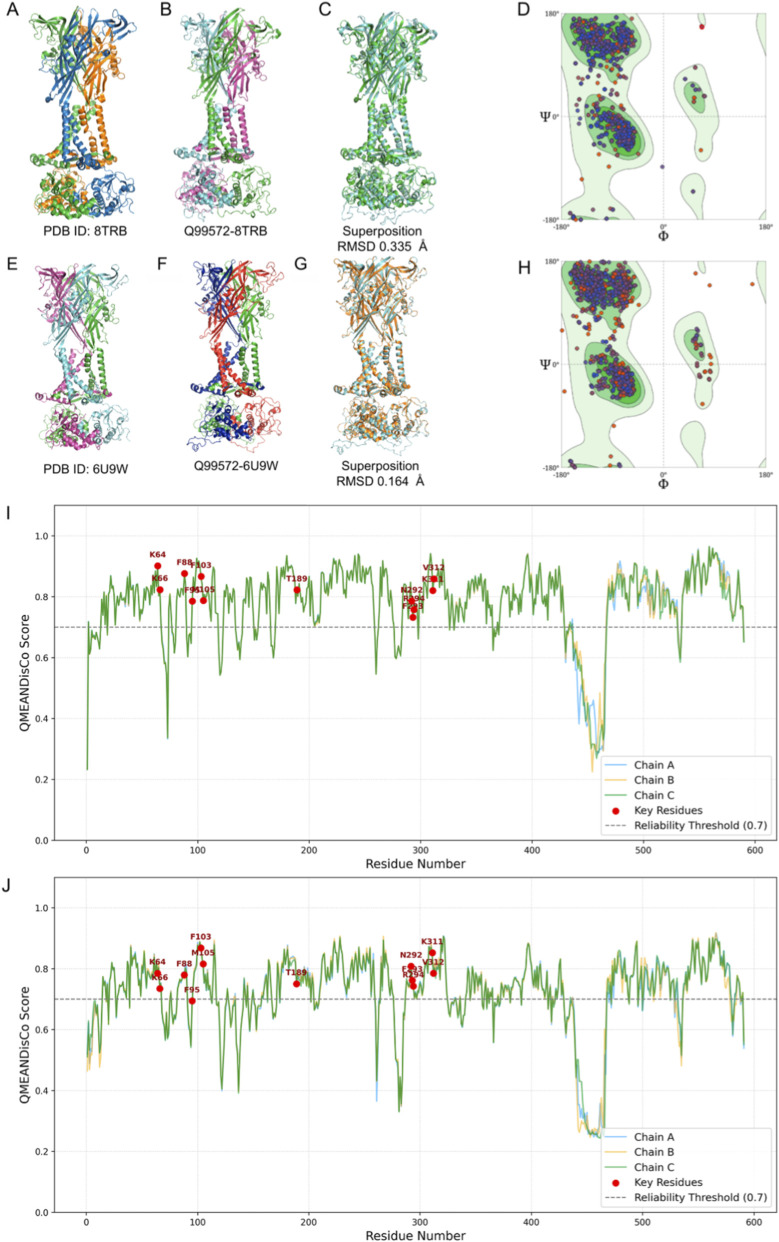
Structural modeling and validation of the hP2X7 receptor using the SWISS-MODEL server with the following templates PDB IDs: 8TRB (rP2X7_closed) and 6U9W (rP2X7_open). **(A)** Structure of 8TRB used as a template for hP2X7 model construction, with its three subunits highlighted in blue, green, and orange. **(B)** Predicted Q99572–8TRB (hP2X7_closed) model generated by SWISS-MODEL, with subunits highlighted in pink, blue, and green. **(C)** Structural superposition between the predicted Q99572–8TRB model (blue) and the 8TRB template structure (orange), yielding a Cα RMSD of 0.335 Å. **(D)** Ramachandran plot of the Q99572–8TRB model. **(E)** Structure of 6U9W used as a template for hP2X7 model construction, with its three subunits highlighted in blue, green, and pink. **(F)** Predicted Q99572–6U9W (hP2X7_open) model generated using SWISS-MODEL, with subunits highlighted in red, blue, and green. **(G)** Structural superposition between the predicted Q99572 -6U9W model (blue) and the 6U9W template structure (orange), yielding a Cα RMSD of 0.164 Å. **(H)** Ramachandran plot of the Q99572–6U9W model, indicating stereochemical quality. **(I,J)** QMEANDisCo per-residue local quality scores for the homology models Q99572–8TRB and Q99572–6U9W, respectively, with individual chains displayed as chain A (blue), chain B (orange), and chain C (green), with key residues highlighted in red.

The 8TRB (rP2X7_closed) and 6U9W (rP2X7_open) structures were used as templates (Figures 1A,E), and the generated models were named Q99572–8TRB (hP2X7_closed) (Figure 1B) and Q99572–6U9W (hP2X7_open) (Figure 1F). Following model construction, structural superposition was performed against the corresponding templates. The Q99572–8TRB model exhibited an RMSD of 0.335 Å relative to 8TRB (Figure 1C), and the Q99572–6U9W model showed an RMSD of 0.164 Å compared to 6U9W (Figure 1G), indicating a high degree of structural similarity.

Stereochemical evaluation through Ramachandran plot analysis demonstrated a high-quality backbone geometry for both models. In the Q99572–8TRB (hP2X7_closed) model, 1,668 residues (94.56%) were located in the most favorable regions, and only 7 residues (0.40%) were in disallowed regions (Figure 1D). Similarly, the Q99572–6U9W (hP2X7_open) model revealed 1,695 residues (95.93%) in the most favored regions and only 11 residues (0.62%) in disallowed regions (Figure 1H). These results indicate that both models exhibit stereochemical quality, with minimal deviations from ideal backbone conformations.

Despite the high overall structural similarity between the generated models and their respective experimental structures, both models exhibited a considerable degree of disorder in the C-terminal region, specifically between residues 440 and 470. This structural ambiguity arises from the absence of corresponding segments in the selected templates, which limits the ability of the modeling algorithm to accurately predict conformations in this region (Figures 1I,J).

Furthermore, the region encompassing residues 282 to 288 exhibited reduced local QMEANDisCo scores, indicating lower structural reliability in this segment. This behavior may be attributed to the substitution of a glutamic acid residue in the template sequence with a valine in the hP2X7 sequence, potentially impacting the local conformation. The global QMEANDisCo scores were 0.77 for the Q99572–8TRB (hP2X7_closed) model and 0.71 for the Q99572–6U9W (hP2X7_open) model, reflecting overall great structural quality and consistency with the experimentally resolved templates.

### 3.2 Construction of hP2X7 models using AF2 and AF3

**FIGURE 2:**
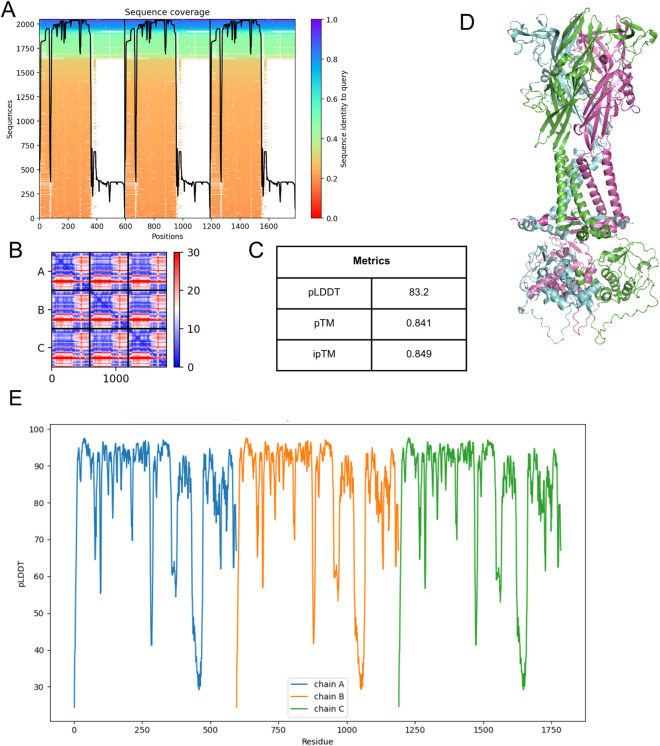
Modeling and validation of the hP2X7 receptor predicted by AF2. **(A)** Sequence coverage over the hP2X7 receptor sequence. **(B)** PAE profile of the generated model. **(C)** AF2 model confidence metrics: pLDDT, pTM, and ipTM. **(D)** Selected multimeter model with the subunits highlighted in green, blue, and pink. **(E)** pLDDT profile by residue in the selected model: pLDDT >90 indicates very high structural confidence, 90< pLDDT >70 indicates high to moderate confidence, and pLDDT <70 indicates low structural confidence.

Structural modeling of the hP2X7 receptor was performed using the multimer version of ColabFold, which is based on AF2. The process yielded five independent models. Analysis of the AF2 database coverage for the P2X7 sequence revealed high coverage up to approximately residue 360, with sequences ranging from 0.2 to 1.0. Notably, only the C-terminal region (residues 360–595) contained sequences with high identity (>0.4) (Figure 2A). All the models presented similar PAE profiles (Figure 2B), with the highest uncertainty concentrated near the C-terminal region. Owing to this consistency, model selection was guided by AF2’s internal confidence metrics: i) pLDDT, which quantifies residue-level confidence; ii) pTM, which indicates global prediction reliability; and iii) ipTM, which assesses the robustness of interchain interfaces in the multimer.

The chosen model presented the highest pTM and ipTM scores (0.841 and 0.840, respectively), along with the second-highest pLDDT score (83.2) (Figures 2C,D). An analysis of pLDDT values by residue in the selected model revealed that most residues exhibited pLDDT scores above 70, indicating high confidence in the predicted structure. However, the C-terminal region is less reliable, with pLDDT scores less than 50 (Figure 2E). This observation aligns with the coverage and PAE results. The full metrics for the other models are available in Supplementary Figure S2.

**FIGURE 3:**
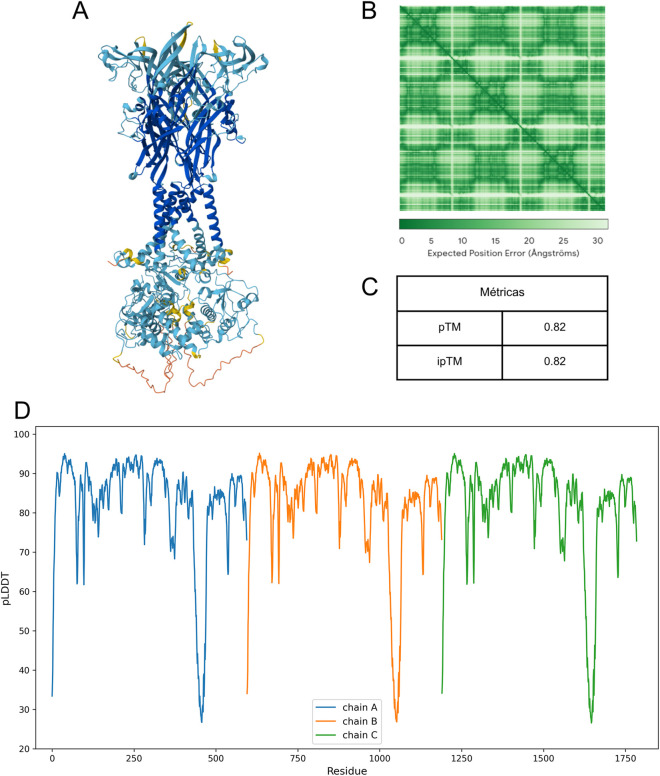
Modeling and validation of the hP2X7 receptor predicted by AF3. **(A)** Structural model of the hP2X7 receptor predicted by AF3. The coloring reflects local confidence values (pLDDT): dark blue (pLDDT >90), light blue (90< pLDDT >70), yellow (70< pLDDT >50), and orange (pLDDT <50), indicating the predicted structural accuracy in different regions of the protein. **(B)** PAE profile of the generated model. **(C)** AF3 model confidence metrics: pLDDT, pTM, and ipTM. **(D)** pLDDT profile by residue in the selected model: pLDDT >90 indicates very high structural confidence, 90< pLDDT >70 indicates high to moderate confidence, and pLDDT <70 indicates low structural confidence.

Modeling with AF3 was conducted using the dedicated server provided by DeepMind, and five models of the hP2X7 receptor were generated at the conclusion of the process. The top-ranked model in AF3 (Figure 3A) showed a PAE similar to that observed in AF2, with a greater predicted error near the C-terminal region (Figure 3B). Furthermore, the model yielded pTM and ipTM scores of 0.82 (Figure 3C), which were slightly lower than those obtained for AF2 (Figure 2C). Residue-level analysis of the pLDDT scores revealed a similar pattern to that of AF2: most residues achieved scores above 70, indicating high confidence in the structural prediction, whereas the C-terminal region remained a zone of lower confidence (Figure 3D). These findings are consistent with results from other assessments, reinforcing that accurate structural modeling in this region continues to pose challenges.

All structural models were superimposed, and their RMSD values were assessed (Table 1). The models generated by AF2, AF3, and Q99572-8TRB (hP2X7_closed) demonstrated higher structural similarity, with RMSD values ranging from 0.748 to 1.429 Å. In contrast, the greatest structural divergence was observed when the models were compared to Q99572-6U9W (hP2X7_open), which was expected given that this structure represents the receptor in its open conformation. These findings further suggest that the models predicted by AF2 and AF3 favor a conformation more closely aligned with the receptor’s closed state.

**TABLE 1:** RMSD (Å) values for structural superposition of the hP2X7 models generated by AF2, AF3, and SWISS-MODEL.

Models	Q99572-AF2	Q99572-AF3	Q99572-8TRB	Q99572-6U9W
Q99572-AF2	0.0	1.008	1.429	2.949
Q99572-AF3	1.008	0.0	0.748	2.484
Q99572-8TRB	1.429	0.748	0.0	4.391
Q99572-6U9W	2.949	2.484	4.391	0.0

In addition, we conducted an in-depth analysis of the orthosteric and allosteric sites of hP2X7 receptor models generated by AF2 and AF3. To this end, we identified key residues involved in ligand binding at these sites based on available literature. According to this information, we assessed the pLDDT scores associated with the corresponding residues in the AF2 and AF3 models, considering both the key residues and those located nearby, to obtain a reliability metric for each site.

To select the residues within the orthosteric site, we referred to the structural study by McCarthy et al. (2019) as a reference, which elucidated the rP2X7 receptor structure using cryo-EM (McCarthy et al., 2019). In this study, the authors compared ATP binding at the orthosteric site of the P2X7 receptor to that of another P2X subclass, highlighting key residues involved in protein-ligand interactions. The residues K64, K66, T189, N292, R294, and K311 are highly conserved across the P2X family.

To evaluate the allosteric site, we referenced the study of Karasawa and Kawate (2016), which utilized X-ray diffraction to investigate the interactions between five allosteric antagonists and the P2X7 receptor (Karasawa and Kawate, 2016). In addition to structurally analyzing ligand-receptor interactions, the authors conducted alanine-scanning mutagenesis experiments to assess residue relevance. Six residues, F88, F95, F103, M105, F293, and V312, were identified as critical for mediating ligand–receptor binding, as their substitution led to significant changes in the IC_50_ values of the ligands.

Analysis of the orthosteric and allosteric sites in the AF2-generated model revealed that the highest average pLDDT scores were observed for residues K311 (93.78) and V312 (93.72), respectively. In contrast, the lowest average pLDDT scores per residue were found for N292 (80.30) in the orthosteric site and F95 (77.73) at the allosteric site (Table 2).

**TABLE 2:** Average pLDDT scores of key and neighboring residues involved in hP2X7 receptor ligand-binding site interactions in models generated by AF2 and AF3.

AlphaFold 2				AlphaFold 3			
Orthosteric site		Allosteric site		Orthosteric site		Allosteric site	
Residue	pLDDT (mean)	Residue	pLDDT (mean)	Residue	pLDDT (mean)	Residue	pLDDT (mean)
K64	90.65	F88	90.65	K64	86.13	F88	81.18
K66	90.94	F95	77.73	K66	84.82	F95	74.12
T189	92.86	F103	85.82	T189	88.06	F103	82.18
N292	80.30	M105	92.00	N292	83.42	M105	86.68
R294	86.87	F293	83.90	R294	84.64	F293	84.25
K311	93.78	V312	93.72	K311	87.68	V312	88.47
Global average	89.23	Global average	87.30	Global average	85.79	Global average	82.82

In the AF3 model, the highest average pLDDT scores per residue were observed for T189 (88.06) and V312 (88.47) in the orthosteric and allosteric sites, respectively. The lowest scores were reported for N292 (83.42) in the orthosteric site and F95 (74.12) in the allosteric site (Table 2).

Furthermore, the global average pLDDT scores per site were higher in the AF2 model (orthosteric: 89.23; allosteric: 87.30) than in the AF3 model (orthosteric: 85.79; allosteric: 82.82). Considering that pLDDT values above 80 indicate regions of high confidence and that values greater than 90 represent very high structural reliability, these results support the conformational robustness of the binding sites, reinforcing their suitability for docking studies.

### 3.3 Docking and interactions

Docking simulations were performed using GNINA, a deep learning–enhanced docking algorithm that integrates CNN scoring functions to improve pose prediction accuracy. The protocol was previously validated through redocking experiments, successfully reproducing the crystallographic binding modes of ATP and JNJ47965567 at the orthosteric and allosteric sites, respectively, with RMSD values below 2.0 Å, thereby confirming the reliability of the docking setup (Supplementary Figure S3).

For the orthosteric site, all receptor model structures were docked to ATP, and the grid definition was based on the coordinates of ATP cocrystallized with the protein structure PDB ID 6U9W (rP2X7_open). The top three poses from each docking run were analyzed for molecular interactions using the PLIP. These analyses focused on identifying interactions involving key residues previously reported in the literature as critical for ligand binding at both orthosteric and allosteric sites.

**FIGURE 4:**
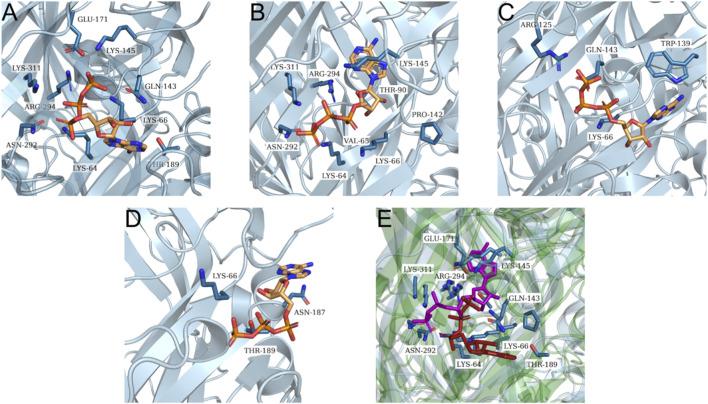
ATP binding at the orthosteric site of the generated hP2X7 receptor models. Representation of molecular interactions between ATP and the different structural models of the hP2X7 receptor following docking simulations. Interactions are shown for the following models: **(A)** Q99572-6U9W; **(B)** Q99572-AF2; **(C)** Q99572-8TRB; and **(D)** Q99572-AF3. **(E)** Superposition of the best docking poses for the experimental model Q99572-6U9W (protein shown in green, ATP in red) and the predicted model Q99572-AF2 (protein shown in blue, ATP in purple), highlighting similarities and differences in ligand positioning and binding site conformation.

The Q99572–6U9W (hP2X7_open) model demonstrated the highest accuracy in predicting ATP binding, which is consistent with expectations given that its template represents the protein in an open-state conformation bound to ATP (Table 3; Figure 4A). The AF2 model exhibited highly relevant interactions within the orthosteric site, with the only notable exception being the interaction with the T189 residue (Figure 4B). In contrast, both the Q99572-8TRB (hP2x7_closed) and AF3 models failed to produce favorable binding poses (Figures 4C,D). The observed discrepancies between the AF2 and AF3 models may be attributed to structural variations within the orthosteric binding site in the latter, as indicated by the RMSD value of 1.008 Å (Figure 4E). Regarding the 8TRB-based model, the lack of suitable interactions is possibly due to conformational distortions introduced by its cocrystallization with an antagonist, an event that may have negatively affected the structural integrity of the orthosteric site. Residues potentially altered by this conformation include F88, F103, Y108, F293, and Y295.

**TABLE 3:** Interactions between the hP2X7 receptor models and key residues involved in ATP binding at the orthosteric site.

Key residues	Q99572-AF2	Q99572-AF3	Q99572-8TRB	Q99572-6U9W
K64	SB	—	—	SB
K66	SB	HB	SB	SB
K145	HB	—	​	SB
T189	—	HB	—	HB
N292	HB	—	—	HB
R294	SB	—	—	SB
K311	SB	—	—	SB

HB, hydrogen bond; SB, salt bridge.

For the docking simulations targeting the allosteric site, all receptor model structures were docked with the JNJ47965567 antagonist. The grid box was defined based on the coordinates of the allosteric ligand JNJ47965567, as determined from its cocrystallized structure PDB ID: 8TRB (rP2X7_closed).

**FIGURE 5:**
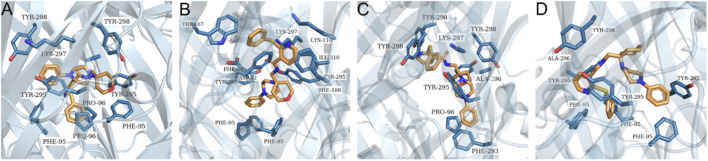
JNJ47965567 binding at the allosteric site of the generated hP2X7 receptor models. Representation of molecular interactions between JNJ47965567 and the different structural models of the hP2X7 receptor following docking simulations. Interactions are shown for the following models: **(A)** Q99572-6U9W; **(B)** Q99572-8TRB; **(C)** Q99572-AF2; and **(D)** Q99572-AF3.

The results of the docking assay demonstrated that the Q99572-6U9W (hP2X7_open) model failed to establish any hydrogen bonds and presented solely hydrophobic interactions (Figure 5A). On the other hand, the Q99572–8TRB (hP2X7_closed) model exhibited the greatest number of expected interactions with the JNJ47965567 ligand (Table 4; Figure 5B), which is consistent with its conformation when bound to the ligand as described in the crystallographic study (Oken et al., 2024). The allosteric binding site was localized in the central region of the protein, which is highly hydrophobic. As a result, all the models displayed primarily hydrophobic interactions. Consequently, all the models exhibited primarily hydrophobic interactions, with their best poses presenting four to five interactions. The AF2 model presented a notable hydrogen bond with residue Y298 (Figure 5C), a key interaction previously reported for this site (Oken et al., 2024). In comparison, the AF3 model formed a hydrogen bond with Y295 (Figure 5D), another essential residue.

**TABLE 4:** Interactions between hP2X7 receptor models and key residues involved in JNJ47965567 antagonist binding at the allosteric site.

Key residues	Q99572-AF2	Q99572-AF3	Q99572-8TRB	Q99572-6U9W
F88	—	—	π-s	—
D92	—	—	HB, HI	—
F95	—	HI	HI	HI
F103	—	—	—	—
M105	—	—	—	—
Y108	—	—	HI, π-s	—
F293	HI	—	—	—
Y295	HI	HI, HB	HI, π-s	HI
K297	HI	—	HB	HI
Y298	HB, π-s	HI	—	HI
I310	—	—	HI	—
A312	—	—	—	—

HB, hydrogen bond; HI, hydrophobic interaction; π-s, π-stacking.

**FIGURE 6:**
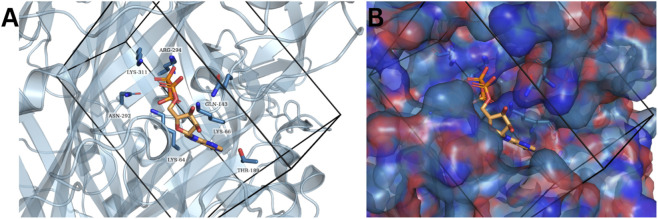
ATP binding at the orthosteric site in the AF3-generated hP2X7 receptor model. **(A)** Interaction map of ATP and the hP2X7 receptor generated using PLIP program. **(B)** Protein-ligand binding pocket. The ATP molecule is depicted in stick format, assuming a characteristic U-shaped conformation oriented toward the interior of the pocket. Binding site regions are color-coded according to their electrostatic charge: marine blue for positively charged areas, red for negatively charged areas, and blue for neutral zones.

Since AF3 can model a protein bound to ions or ligands, we analyzed the generated hP2X7 receptor model by inputting this protein sequence along with three ATP molecules as ligands. Interaction analysis revealed that the generated model exhibited a U-shaped conformation, as previously described in the literature (Hattori and Gouaux, 2012; Jiang et al., 2021), and revealed hydrogen bonding interactions involving residues K64, K66, T189, N292, R294, and K311 (Figures 6A,B).

Although the results are encouraging, the binding orientation of ligands within the protein’s active site appeared slightly misaligned. According to Jiang and colleagues (2021), residue K64 forms hydrogen bonds with all three ATP phosphate groups (Jiang et al., 2021). However, in the generated model, this role was instead performed by residue K66. Additionally, interactions between γ-phosphate and residues K64, N292, N294, and K311 have been previously reported, but in our model, no bond was observed between γ-phosphate and K311 (Figures 6A,B). It is also important to consider that intermolecular interactions between rat and human P2X7 receptors vary substantially. Therefore, only the elucidation of the interaction between the hP2X7 receptor cocrystallized with ATP can confirm the differences in intermolecular binding across these systems.

## 4 Discussion

Recent advances in artificial intelligence have revolutionized structural biology, enabling highly accurate protein modeling with minimal experimental input. These emerging methodologies are especially valuable for studying the human P2X7 purinergic receptor, a clinically important target for which crystallographic data are scarce. In this context, we sought to systematically compare artificial intelligence (AI)-based structural predictions with those of traditional modeling strategies.

To evaluate the accuracy of structural models generated by neural networks, such as AlphaFold, compared with conventional modeling approaches, we adopted distinct strategies to model the hP2X7 receptor. As an initial step, two reference models were built using comparative modeling on the SWISS-MODEL server (Waterhouse et al., 2018). This platform was strategically selected given that the hP2X7 receptor has experimental templates with high coverage, sequence identity, and, crucially, high resolution (below 3 Å), despite being a large trimeric complex with nearly 1800 residues. Such conditions are ideal for homology-based modeling, enabling the generation of highly reliable structural models that serve as robust benchmarks for assessing the performance of AI-based prediction methods.

Models were constructed using two rP2X7 receptor proteins extracted from the PDB, 8TRB (rP2X7_closed) and 6U9W (rP2X7_open), as templates, which exhibit approximately 100% coverage, approximately 80% sequence identity, and high resolution. The resulting models, Q99572-8TRB (hP2X7_closed) and Q99572-6U9W (hP2X7_open), showed high global reliability metrics, with QMEANDisCo scores of 0.77 and 0.71, respectively, and RMSD values of 0.335 Å and 0.164 Å, respectively, compared with their corresponding experimental templates. Both models demonstrated elevated structural disorder in the C-terminal region, as revealed by per-residue confidence analysis. This disorder is correlated with the absence of template coverage for residues 440–470.

Additionally, the Q99572-6U9W (hP2X7_open) model exhibited greater variability between residues 282–288, possibly due to the substitution of glutamic acid with valine at position 285. Ramachandran plot analysis revealed that approximately 95% of the residues are positioned in favored regions. The Q99572-8TRB (hP2X7_closed) model presented 0.40% outliers (7 residues), all of which were located within the intracellular domain, whereas the Q99572-6U9W (hP2X7_open) model exhibited 0.62% outliers, with residue V285 being the only exception outside the intracellular region. Collectively, these findings suggest that both models are highly reliable (QMEANDisCo >0.7), particularly within the extracellular domain, which is a critical region for functional and structural characterization.

We then modeled the hP2X7 receptor using AF2 and AF3. The structure generated by AF2 exhibited high local reliability (pLDDT: 83.2), robust global confidence (pTM: 0.841), and high interchain prediction accuracy (ipTM: 0.849). Similarly, the AF3 model demonstrated consistent confidence scores, with a pTM of 0.82 and an ipTM of 0.82.

As observed in the comparative modeling approach, both AlphaFold models exhibited pronounced structural disorder in the C-terminal region, particularly across residues 440 to 470, as indicated by a lower pLDDT. Sequence coverage analysis performed with AF2 also confirmed reduced coverage in this segment compared with the rest of the protein. Despite analogous declines in per-residue validation metrics (pLDDT and QMEANDisCo) between residues 440–470, the AlphaFold models demonstrated more pronounced disorder in this region, which was projected outward from the protein and adopted a loop-like conformation. This likely reflects a form of model hallucination (Abramson et al., 2024), a known limitation of AlphaFold when predicting extended loop regions (Bertoline et al., 2023; Binbay et al., 2023).

To determine the conformational state predicted by the AF2 and AF3 models, their structures were superimposed onto P2X7 receptor structures obtained through comparative modeling, encompassing both open and closed conformations. Structural analysis revealed that both the AF2 and the AF3 models showed greater structural similarity to the closed state, with RMSD values of 1.429 Å and 0.748 Å, respectively. These results align with expectations, considering that few structures of the P2X7 receptor in the open state has been experimentally determined, whereas more than 20 structures in the closed state have been resolved and incorporated into the AlphaFold training databases.

A current limitation of both AF2 and AF3 is their inability to predict multiple conformational states. The models are restricted to generating the most likely structure based on the training data, without allowing the selection between alternative states such as the open or closed conformations (Bertoline et al., 2023). Consequently, the only viable strategy for modeling the hP2X7 receptor in the open state remains comparative modeling.

In addition to assessing the conformational states predicted by the AlphaFold models, we also investigated their active sites to evaluate their reliability for docking studies. First, we analyzed the pLDDT scores of key residues involved in ligand interactions with the hP2X7 receptor, along with their neighboring regions in both the orthosteric binding pocket and the allosteric binding pocket. These confidence scores serve as indicators of structural accuracy, particularly compared to experimentally resolved structures. It is important to note that AlphaFold predictions are valuable hypotheses and accelerate, but do not replace, experimental structure determination.

The results indicated that the AF2 model yielded higher pLDDT values at the orthosteric (89.23) and allosteric (87.30) sites, including both the key residues and their surroundings, than did the AF3 model, which scored 85.79 and 82.82, respectively. Notably, the AF2 model outperforms AF3 in validation metrics (pLDDT, pTM, and ipTM scores) and demonstrated better overall performance across the binding sites, despite the substantial advancements reported in the AF3 publication relative to the multimodel version of AF2 (Abramson et al., 2024).

Finally, we assessed model performance in docking experiments using hP2X7 receptor models generated using comparative modeling, i.e., Q99572-8TRB (hP2X7_closed), Q99572-6U9W (hP2X7_open), and AF2 and AF3. Docking simulations occurred at both the orthosteric and allosteric sites. ATP was used as the orthosteric site ligand, whereas JNJ47965567 was chosen for the allosteric site (Karasawa and Kawate, 2016; Jiang et al., 2021). Docking simulations were conducted using GNINA, a deep learning-based software that employs CNN to predict ligand binding poses and affinities. Postdocking and intermolecular interactions between the ligands and key residues of the hP2X7 receptor were characterized using the PLIP platform.

Docking assays at the orthosteric site revealed that the Q99572-6U9W (hP2X7_open) model exhibited all the predicted interactions with key residues K64, K66, K145, T189, N292, R294, and K311 involved in ATP binding, which is consistent with the literature findings (Caseley et al., 2015; McCarthy et al., 2019; Jiang et al., 2021). The AF2-generated model produced comparable results, with the notable exception of the missing hydrogen bond involving residue T189. In contrast, the AF3 model displayed only two interactions with critical residues involved in ATP binding, representing a substantial deviation from the performance of the AF2 model. Moreover, the Q99572-8TRB (hP2X7_closed) model showed only one interaction, possibly reflecting conformational disparities between this structure and Q99572-6U9W (hP2X7_open), according to the RMSD of 4.391 Å.

Docking simulations at the allosteric site revealed that the Q99572-8TRB (hP2X7_closed) model formed hydrophobic interactions with residues F88, F95, Y295, and I310, along with hydrogen bonds involving D92 and K297. These findings align with the known binding profile of the ligand JNJ47965567, as described in previous studies (Karasawa and Kawate, 2016; Oken et al., 2024). In comparison, both the AF2 and AF3 models presented three hydrophobic interactions and one hydrogen bond, involving critical residues for ligand recognition and binding. Conversely, the Q99572-6U9W (hP2X7_open) model lacked hydrogen bonding entirely and displayed only hydrophobic contacts between the ligand and receptor.

Importantly, significant structural differences exist between the closed and open conformational states of the hP2X7 receptor, particularly within its ligand-binding regions. The binding of ATP at the orthosteric site induces pronounced conformational rearrangements, including the approximation of positively charged residues that interact with the triphosphate moiety of ATP, such as K64, K66, and K311, among others. These electrostatic interactions contribute to the stabilization of the activated state. In addition, ATP binding promotes substantial structural displacement of the second transmembrane helix (TM2), which is directly associated with channel opening and ion permeation (McCarthy et al., 2019).

The allosteric binding sites are located adjacent to the orthosteric site (Karasawa and Kawate, 2016). The association of allosteric antagonists with these sites prevents channel opening by interfering with the conformational changes required for activation (Karasawa and Kawate, 2016). Notably, ATP-induced conformational changes reduce the accessibility and volume of the allosteric cavity. This was demonstrated through cysteine-scanning mutagenesis experiments in which ATP binding prevented the access of a large cysteine-reactive agent to engineered cysteine residues within the allosteric region. These findings indicate that ATP binding induces a conformational contraction that sterically restricts the allosteric pocket. Conversely, binding of allosteric ligands does not appear to prevent orthosteric ATP binding, as evidenced by the crystal structure of pdP2X7 cocrystallized with both ATP and the allosteric antagonist A804598 (PDB ID: 5U2H) (Karasawa and Kawate, 2016).

From a structure-based drug design perspective, these conformational differences have important implications. The identification of allosteric antagonists should preferentially employ receptor structures in the closed state, where the allosteric cavity is more accessible. In contrast, the screening of competitive orthosteric antagonists should rely on models representing the ATP-bound open conformation, which accurately reflects the activated binding environment. Interestingly, the structures predicted by AlphaFold, particularly those generated by AF2, display conformational features resembling the closed state of the hP2X7 receptor. However, similar to the PDB ID 5U2H structure, these models also demonstrated sensitivity in docking assays targeting the orthosteric site, suggesting partial structural compatibility with both functional states.

A promising approach for evaluating protein-ligand interactions has been integrated into AF3, which now supports the modeling of proteins in complex with ions and ligands. To explore the potential of this tool in the context of the hP2X7 receptor, we performed joint modeling of the receptor protein with three ATP molecules as ligands. The results were encouraging: the generated model presented hydrogen bonds with residues K64, K66, T189, N292, R294, and K311 and adopted a U-shaped conformation as described in the literature (Hattori and Gouaux, 2012; Jiang et al., 2021). A recent study also highlighted the ability of AF3 to accurately model protein-ligand complexes, surpassing the quality of results obtained through docking assays, although certain limitations remain (Zheng et al., 2025).

Several studies have aimed to identify novel selective inhibitors of the P2X7 receptor using VS, a computational approach for discovering potential ligands for biological targets that are often paired with docking (Alberto et al., 2020). Owing to the lack of experimental structures for the hP2X7 receptor, the development of accurate computational models capable of simulating the protein’s biological context is essential (Scardino et al., 2023).

Caseley et al. (2016) employed comparative modeling to construct an hP2X7 receptor model based on the structure of zebrafish P2X4. This study identified three compounds with inhibitory activity, including C60, which selectively blocked pore opening (Caseley et al., 2016). Pasqualetto et al. (2023) performed VS assays aiming to find orthosteric inhibitors for P2X4 and discovered a compound (gp-25) with inhibitory activity on the hP2X7 receptor (Pasqualetto et al., 2023). Fresta et al. (2020) constructed an hP2X7 receptor model using an rP2X7 structure (PDB ID: 6U9V) and identified the compound DHTS through VS, which demonstrated a protective effect on the blood‒retinal barrier after stimulation with high glucose and BzATP (Fresta et al., 2020).

Zhao et al. (2021) developed an hP2X7 receptor model based on the pdP2X7 structure (PDB ID: 5U2H) and, through VS, identified three compounds with experimentally confirmed inhibitory activity on the receptor (Zhao et al., 2021). Ferreira et al. (2024) constructed a model of the pdP2X7 receptor and applied a hybrid approach combining shape-based analysis and docking to identify novel antagonists derived from natural products. Eight compounds were proposed to have a predicted affinity for the pdP2X7 receptor (Ferreira et al., 2024). Zuanon et al. (2025) applied an hP2X7 receptor model based on the pdP2X7 receptor structure (PDB ID: 5U1X), along with the aforementioned hybrid methodology, to screen novel antagonists in commercial databases. Their VS efforts led to the identification of two compounds that demonstrated high experimental inhibitory activity on the hP2X7 receptor (Zuanon et al., 2025).

Despite continued efforts to identify novel selective antagonists of the P2X7 receptor, no drugs have yet been approved for clinical use. In this context, attention-based AI tools such as AlphaFold have the potential to drive significant advances in the field. Our models generated with AF2 and AF3 showed structural similarity and confidence metrics comparable to those obtained through comparative modeling. Moreover, docking simulations using the AF2-generated model revealed strong interactions with ATP at the orthosteric site and favorable binding with JNJ47965567 at the allosteric site. These findings support the suitability of the model for VS targeting both binding sites.

Despite current limitations, such as the inability to select different conformational states, which can significantly affect docking and VS accuracy (Scardino et al., 2023), refinement methods such as induced-fit-based protocol (IFD-MD) and bAles demonstrate strong potential in mitigating these constraints (Zhang et al., 2023; Sen et al., 2025). Furthermore, the enhanced ligand-protein prediction capabilities introduced by AF3 may revolutionize the execution of VS assays, playing a pivotal role in accelerating drug discovery.

## 5 Limitations

This study presents some limitations that should be acknowledged. First, the docking validation analyses were restricted to two ligands: ATP at the orthosteric site and JNJ47965567 at the allosteric site. Although these ligands are well characterized and structurally validated in crystallographic complexes, a broader evaluation would be necessary to assess the predictive performance of the AlphaFold-derived models more comprehensively. Future investigations should expand the ligand set to include additional experimentally validated antagonists, as well as structurally diverse compound libraries, including decoy sets, to evaluate robustness, selectivity, and enrichment performance in VS scenarios.

Second, the present study relied exclusively on docking simulations. While docking provides valuable insights into binding pose prediction and potential interaction patterns, it does not account for receptor flexibility beyond the static conformations employed. No molecular dynamics (MD) simulations were performed to assess the stability of the predicted ligand–receptor complexes over time or to refine binding modes through conformational sampling. The incorporation of MD simulations in future studies would allow the evaluation of binding stability, dynamic conformational changes, and a more reliable estimation of binding affinities through methods such as Molecular Mechanics/Poisson–Boltzmann surface area or related free-energy approaches.

Together, these limitations highlight that the present findings represent an initial structural and docking-based assessment, which should be complemented by dynamic and thermodynamic analyses in subsequent investigations.

## 6 Conclusion

This study aimed to evaluate the applicability of attention-based neural network modeling tools, such as AF2 and AF3, in comparison to conventional comparative modeling approaches. Both approaches successfully produced high-quality models of the hP2X7 receptor. All the compounds exhibited notable structural disorder in the C-terminal region, primarily due to the absence of residues 440–470 in the template structures. While both techniques have limitations, the AlphaFold-generated models showed more pronounced distortions in this region, likely resulting from “hallucinations” caused by sparse data coverage. Furthermore, the AlphaFold models displayed greater structural similarity to the closed conformation of the hP2X7 receptor, suggesting a potential bias of the algorithm toward this state. This may be attributed to the greater availability of experimentally resolved structures of the P2X7 receptor in its closed form.

Confidence analysis of the AlphaFold-generated models revealed that the model produced by AF2 exhibited higher scores than its AF3 counterpart. Docking simulations further corroborate that the AF2 model demonstrated a greater number of interactions with key residues in the orthosteric and allosteric binding sites than the AF3 model. These findings suggest that the AF2-derived hP2X7 receptor model may serve as a suitable structural framework for VS studies targeting both orthosteric and allosteric sites of the receptor, potentially contributing to the development of improved VS protocols aimed at identifying novel P2X7 receptor antagonists. Nevertheless, conventional receptor modeling based on experimentally resolved templates representing distinct conformational states (open and closed) remains the gold standard. Such approaches allow the receptor to be explicitly modeled in functionally relevant conformations, which is particularly important when searching for orthosteric or allosteric ligands that preferentially bind to specific activation states of the P2X7 receptor.

Additionally, AF3 demonstrated the ability to model an hP2X7 receptor–ATP complex in which it is possible to visualize the interaction between ATP and the orthosteric site involving key binding residues, which is consistent with the characteristic “U-shaped” conformation of the ligand reported in the literature. We anticipate that further improvements to protein modeling could revolutionize the execution of VS workflows aimed at discovering novel P2X7 receptor antagonists.

## Data Availability

The original contributions presented in the study are included in the Supplementary Material, and further inquiries can be directed to the corresponding author.
