# Supplementary material for: Modeling the P2X7 receptor: a comparative analysis of conventional methods and AlphaFold
Source: Front Pharmacol. 2026 Apr 21;17:1685425. doi: 10.3389/fphar.2026.1685425 (PMC13139673; doi:10.3389/fphar.2026.1685425)
Supplement: Supplementary file 1 [file DataSheet1.pdf]

Supplementary Material

Supplementary Table 1. P2X7 antagonists in clinical studies

P2X7 antagonist	Pharmaceutical company	Phase	Condition	Status	Clinical trial ID
EVT-401	Evotec	1	Rheumatoid arthritis	Completed (2009)	-
CE-224,535	Pfizer	2	Rheumatoid arthritis	Completed (2009)	NCT00628095
AZD9056	AstraZeneca	2	Rheumatoid arthritis	Completed (2013)	NCT00520572
GSK14822160	GlaxoSmithKline	1	Chronic inflammatory pain	Completed (2017)	NCT00849134
JNJ-54175446	Johnson & Johnson	2	Depression	In progress (2025)	NCT04116606
JNJ-55308942	Johnson & Johnson	2	Bipolar depression	Completed (2025)	NCT05328297

Supplementary Table 2. Structures found as potential templates for comparative modeling of the human P2X7 receptor (UniProt ID: Q99572)

PDB ID	Max score	Total score	Query cover	E-value	Identity
6U9V	983	983	100%	0.0	80.34%
6U9W	893	893	100%	0,0	80.34%
8TRB	982	983	100%	0.0	80.34%
5U1L	627	627	57%	0.0	85.84%
5XW6	361	361	56%	1e-120	50.75%
8JV5	321	321	58%	1e-104	46.72%
8JV6	320	320	58%	2e-104	46.86%
4DW0	315	315	57%	3e-102	46.51%
3H9V	314	314	57%	6e-102	46.51%
4DW1	313	313	57%	6e-102	46.51%

```
Q99572      MPACCSCSDVFQYETNKVTRIQSMNYGTIKWFFHVIIFSYVCFALVSDKLYQRKEPVISS     60
8TRB        MPACCSWNDVFQYETNKVTRIQSVNYGTIKWILHMTVFSYVSFALMSDKLYQRKEPLISS     60
6U9W        MPACCSWNDVFQYETNKVTRIQSVNYGTIKWILHMTVFSYVSFALMSDKLYQRKEPLISS     60
            ****** .***************:*******::*: :****.***:**********:***

Q99572      VHTKVKGIAEVKEEIVENGVKKLVHSVFDTADYTFPLQGNSFFVMTNFLKTEGQEQRLCP     120
8TRB        VHTKVKGVAEVTENVTEGGVTKLVHGIFDTADYTLPLQGNSFFVMTNYLKSEGQEQKLCP     120
6U9W        VHTKVKGVAEVTENVTEGGVTKLVHGIFDTADYTLPLQGNSFFVMTNYLKSEGQEQKLCP     120
            *******:***.*::.*.**.****.:*******:************:**:*****:***

Q99572      EYPTRRTLCSSDRGCKKGWMDPQSKGIQTGRCVVYEGNQKTCEVSAWCPIEAVEEAPRPA     180
8TRB        EYPSRGKQCHSDQGCIKGWMDPQSKGIQTGRCIPYDQKRKTCEIFAWCPAEEGKEAPRPA     180
6U9W        EYPSRGKQCHSDQGCIKGWMDPQSKGIQTGRCIPYDQKRKTCEIFAWCPAEEGKEAPRPA     180
            ***:* . * **:** ****************: *: ::****: **** *  :******

Q99572      LLNSAENFTVLIKNNIDFPGHNYTTRNILPGLNITCTFHKTQNPQCPIFRLGDIFRETGD     240
8TRB        LLRSAENFTVLIKNNIDFPGHNYTTRNILPGMNISCTFHKTWNPQCPIFRLGDIFQEIGE     240
6U9W        LLRSAENFTVLIKNNIDFPGHNYTTRNILPGMNISCTFHKTWNPQCPIFRLGDIFQEIGE     240
            **.****************************:**:****** *************:* *:

Q99572      NFSDVAIQGGIMGIEIYWDCNLDRWFHHCRPKYSFRRLDDKTTNVSLYPGYNFRYAKYYK     300
8TRB        NFTEVAVQGGIMGIEIYWDCNLDSWSHRCQPKYSFRRLDDKYTNESLFPGYNFRYAKYYK     300
6U9W        NFTEVAVQGGIMGIEIYWDCNLDSWSHRCQPKYSFRRLDDKYTNESLFPGYNFRYAKYYK     300
            **::**:**************** * *:*:*********** ** **:************

Q99572      ENNVEKRTLIKVFGIRFDILVFGTGGKFDIIQLVVYIGSTLSYFGLAAVFIDFLIDTYSS     360
8TRB        ENGMEKRTLIKAFGVRFDILVFGTGGKFDIIQLVVYIGSTLSYFGLATVCIDLIINTYAS     360
6U9W        ENGMEKRTLIKAFGVRFDILVFGTGGKFDIIQLVVYIGSTLSYFGLATVCIDLIINTYAS     360
            **.:*******.**:********************************:* **::*:**:*

Q99572      NCCRSHIYPWCKCCQPCVVNEYYYRKKCESIVEPKPTLKYVSFVDESHIRMVNQQLLGRS     420
8TRB        TCCRSRVYPSCKCCEPCAVNEYYYRKKCEPIVEPKPTLKYVSFVDEPHIWMVDQQLLGKS     420
6U9W        TCCRSRVYPSCKCCEPCAVNEYYYRKKCEPIVEPKPTLKYVSFVDEPHIWMVDQQLLGKS     420
            .****::** ****:**.*********** **************** ** **:*****:*

Q99572      LQDVKGQEVPRPAMDFTDLSRLPLALHDTPPIPGQPEEIQLLRKEATPRSRDSPVWCQCG     480
8TRB        LQDVKGQEVPRPQTDFLELSRLSLSLHHSPPIPGQPEEMQLLQIEAVPRSRDSPDWCQCG     480
6U9W        LQDVKGQEVPRPQTDFLELSRLSLSLHHSPPIPGQPEEMQLLQIEAVPRSRDSPDWCQCG     480
            ************  ** :**** *:**.:*********:***: **.******* *****

Q99572      SCLPSQLPESHRCLEELCCRKKPGACITTSELFRKLVLSRHVLQFLLLYQEPLLALDVDS     540
8TRB        NCLPSQLPENRRALEELCCRRKPGQCITTSELFSKIVLSREALQLLLLYQEPLLALEGEA     540
6U9W        NCLPSQLPENRRALEELCCRRKPGQCITTSELFSKIVLSREALQLLLLYQEPLLALEGEA     540
            .********.:*.*******:*** ******** *:****..**:***********: ::

Q99572      TNSRLRHCAYRCYATWRFGSQDMADFAILPSCCRWRIRKEFPKSEGQYSGFKSPY-----     595
8TRB        INSKLRHCAYRSYATWRFVSQDMADFAILPSCCRWKIRKEFPKTQGQYSGFKYPY-----     595
6U9W        INSKLRHCAYRSYATWRFVSQDMADFAILPSCCRWKIRKEFPKTQGQYSGFKYPYSNSAV     600
             **:*******.****** ****************:*******::******* **

Q99572      ---------   595
8TRB        ---------   595
6U9W        DAGLEVLFQ   609
```

Supplementary Figure 1. Amino acid sequence alignment of proteins Q99572 (hP2X7), 6U9W (rP2X7_open), and 8TRB (rP2X7_closed). The alignment of these protein sequences was performed using the CLUSTAL Omega server. In the alignment output, asterisks (*) denote fully conserved positions, where amino acid residues are identical across all sequences. Colons (:) indicate substitutions with a high degree of conservation, while periods (.) represent substitutions with lower conservation. Hyphens (–) mark gaps introduced during alignment, and blank spaces correspond to nonconserved positions.

A

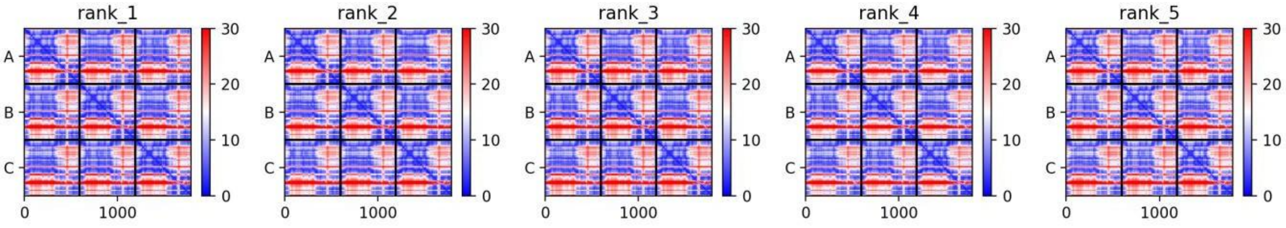
rank_1
rank_2
rank_3
rank_4
rank_5
A
B
C
0
1000
30
20
10
0

B

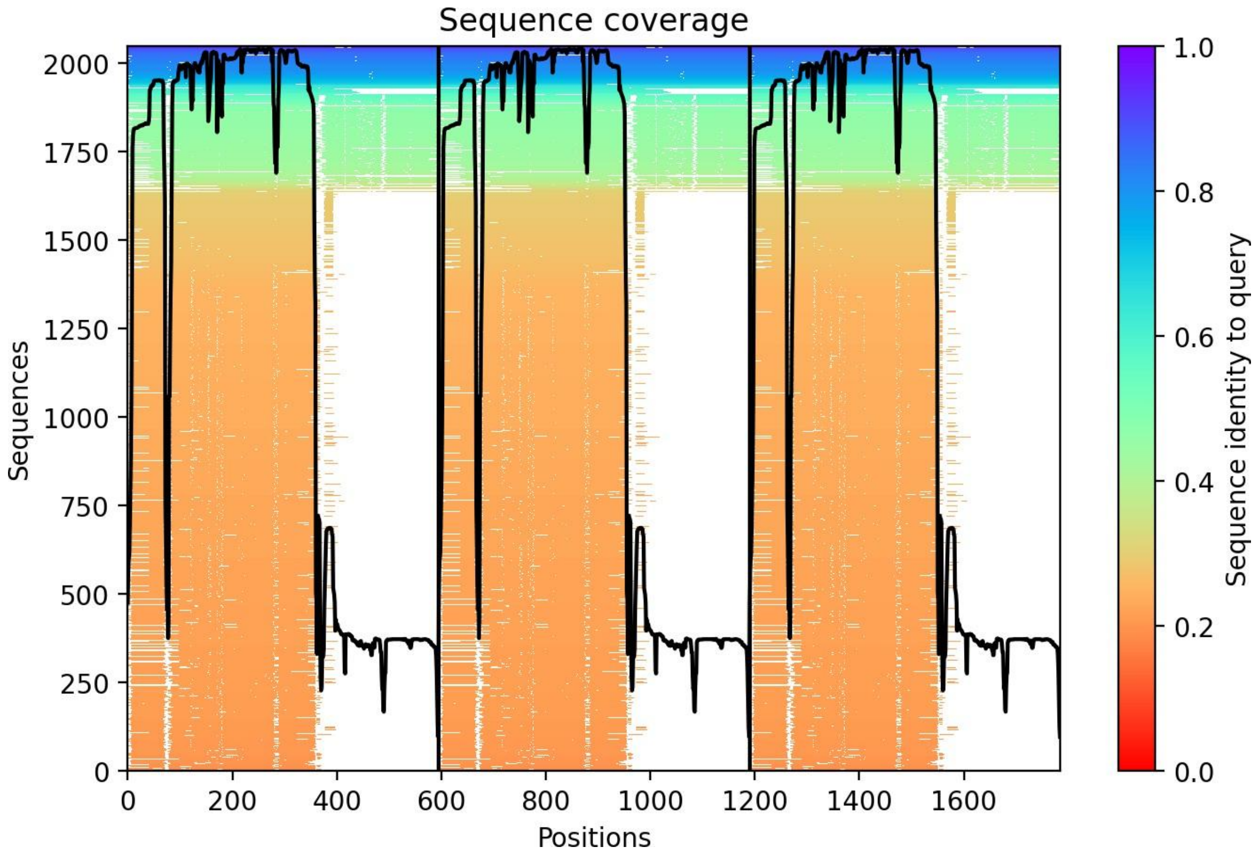
Sequence coverage
Sequences
Positions
Sequence identity to query
0
250
500
750
1000
1250
1500
1750
2000
0
200
400
600
800
1000
1200
1400
1600
0.0
0.2
0.4
0.6
0.8
1.0

C

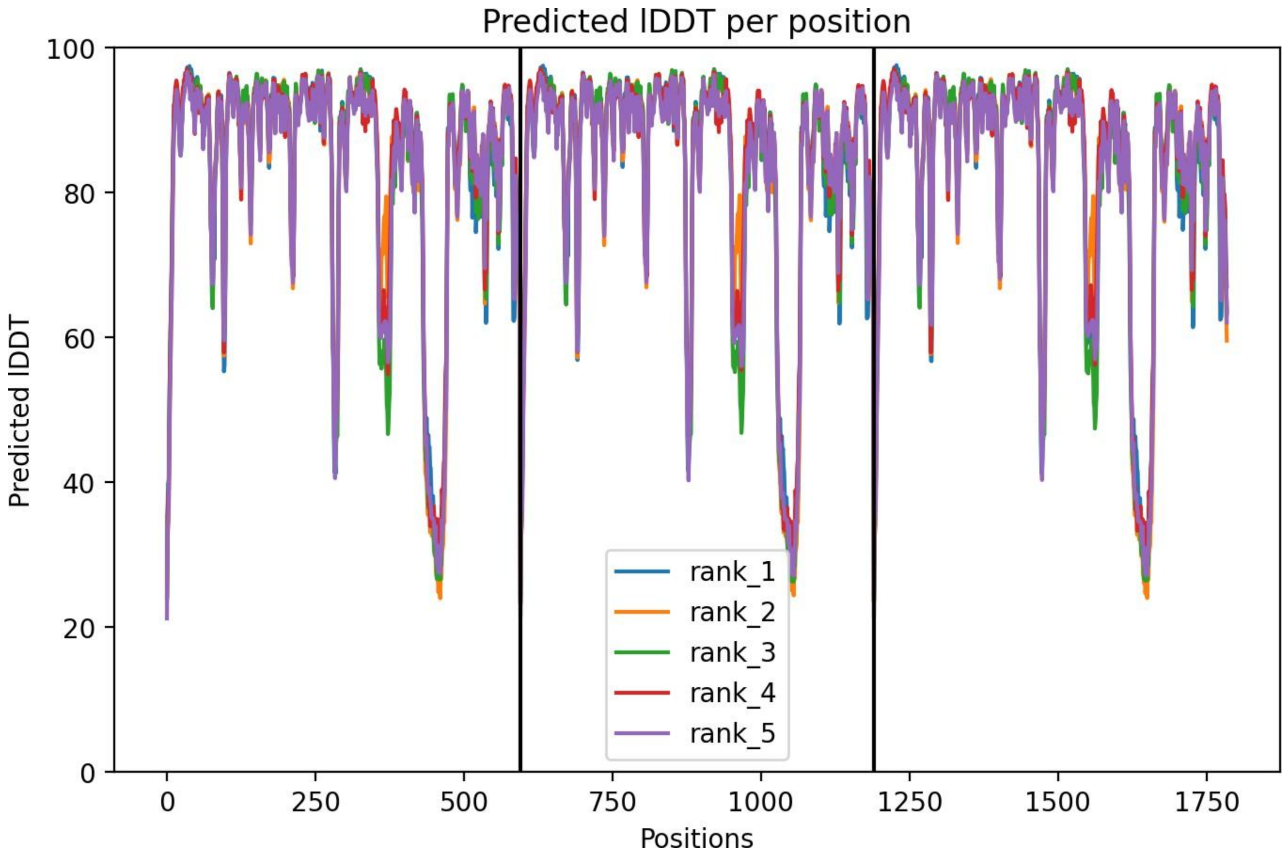

Supplementary Figure 2. Confidence metrics for the five models obtained in AlphaFold2. (A) PAE profile. (B) Sequence coverage over the P2X7 receptor sequence. (C) pLDDT profile by residue in the models.

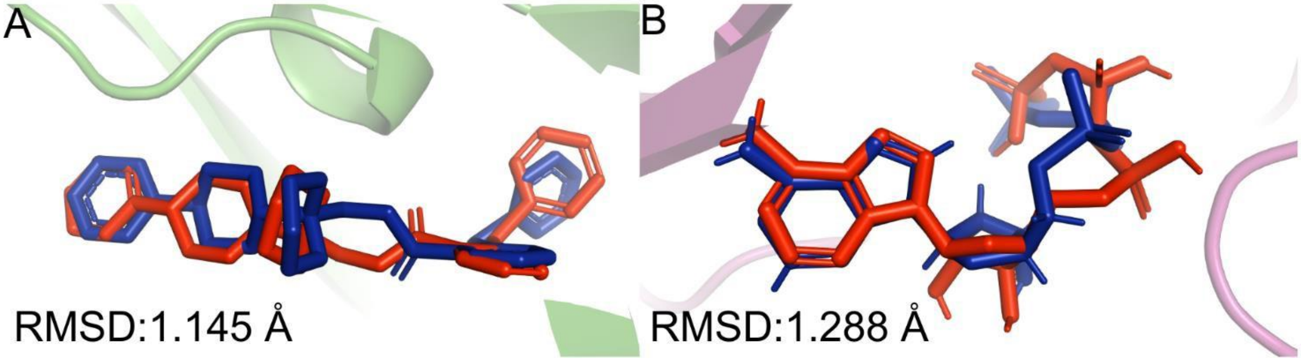

Supplementary Figure 3. Redocking validation of the orthosteric and allosteric binding sites of the P2X7 receptor. Redocking simulations were performed using GNINA v1.3 with the rat P2X7 structures PDB ID: 8TRB (closed conformation, cocrystallized with JNJ47965567) and PDB ID: 6U9W (open conformation, cocrystallized with ATP). (A) Allosteric site redocking using 8TRB: the P2X7 structure is shown in green, the experimental pose of JNJ47965567 in blue, and the GNINA-predicted pose in red. Structural superposition yielded an RMSD of 1.145 Å. (B) Orthosteric site redocking using 6U9W: the P2X7 structure is shown in pink, the experimental ATP pose is shown in blue, and the GNINA-predicted pose is shown in red. Structural superposition yielded an RMSD of 1.288 Å.
